# Diets Rich in *n*-6 or *n*-3 Polyunsaturated Fatty Acids Combined with Exercise Influences Tumour Development in a Her-2 Breast Cancer Model

**DOI:** 10.3390/nu18142262

**Published:** 2026-07-10

**Authors:** Lyn M. Hillyer, Aritra Bhattacharjee, Jessie L. Burns, William J. Muller, Lindsay E. Robinson, David W. L. Ma

**Affiliations:** 1 Department of Human Health Sciences, University of Guelph, Guelph, ON N1G 2W1, Canada; lhillyer@uoguelph.ca (L.M.H.); a.bhattacharjee@mail.utoronto.ca (A.B.); jessielburns-phd@outlook.com (J.L.B.); 2 Department of Biochemistry, Rosalind and Morris Goodman Cancer Centre, McGill University, Montreal, QC H3A 1A3, Canada; william.muller@mcgill.ca

**Keywords:** polyunsaturated fat, exercise, breast cancer, mammary tumour

## Abstract

**Background/Objectives**: Dietary changes or increased physical activity to promote health accompanies breast cancer treatment to improve outcomes and prevent cancer recurrence. However, primary breast cancer prevention studies are limited and while there is a growing benefit to diet and exercise, the combinatory benefits of diet and exercise are limited. Therefore, the objective of this exploratory study was to determine the effects of exposure to diets high in *n*-3 or *n*-6 polyunsaturated fatty acids (PUFA) combined with exercise on mammary tumour development in female MMTV-neu(ndl)YD5 mice. **Methods**: Animals were fed maternal diets containing 10% safflower oil (*n*-6 PUFA-enriched) or 3% menhaden oil + 7% safflower oil (*n*-3 PUFA enriched) for 20 weeks (wks). Within diets, mice were further divided into sedentary control or exercise groups (n = 8–12). Exercised mice were run on a treadmill from 8 to 12 wks of age before tumours developed. Once tumours were detected, volume and multiplicity were measured throughout the study. **Results**: Mice consuming *n*-3 PUFA had reduced tumour volume by 55% (*p* < 0.001) and weight by 42% (*p* = 0.02) compared to mice fed *n*-6 PUFA. Exercised *n*-3 PUFA-fed mice experienced 10% longer tumour-free status (TD50) than their sedentary counterparts and 24% longer TD50 than sedentary mice fed the *n*-6 PUFA enriched diet (*p* = 0.001). Exercised *n*-6 PUFA-fed mice established tumours 11% sooner than their sedentary counterparts and developed the largest and greatest number of tumours throughout the study (*p* < 0.0001). **Conclusions**: Overall, diet quality plays a significant role in mammary tumour outcomes. The effect of exercise on tumour outcomes was not as clear, but this exploratory study highlights the need to consider the temporal trajectory of tumour development that impacts not only lifespan but healthspan.

## 1. Introduction

Breast cancer (BC) is among the top three most commonly diagnosed cancers and is the leading cause of cancer death in women globally [1]. Exercise and diet are major modifiable risk factors. An estimated 30–50% of BC cases can be prevented by lifestyle modifications including consumption of a healthy diet and regular exercise [2]. There is an abundance of research indicating *n*-6 and *n*-3 PUFA promote or attenuate BC outcomes, respectively [3,4,5,6,7,8,9]. Epidemiological evidence shows an association between dietary fat consumption and BC risk. For instance, Asian populations consuming diets rich in long-chain *n*-3 PUFA found in fatty fish have a decreased risk of developing BC [3,4]. In contrast, Western populations consuming diets high in *n*-6 PUFA, found in vegetable oils, and saturated fatty acids (SFA) found in meat and dairy products, have an increased risk of BC [5,8]. It is also important to account for the timing of dietary fat exposure as maternal and pre-pubertal exposure to dietary fats throughout critical points of mammary gland development may affect BC risk later in life [6,9]. Consequently, to evaluate long-term effects on BC risk, studies should also consider potential exposures beginning in utero.

In addition to diet, previous research has shown exercise can mitigate tumorigenesis, at all stages of BC, including prevention, treatment and post-treatment care [7]. A 2016 meta-analysis showed physically active women had a 12–21% lower risk of BC than sedentary women [10]. A recent study revealed participating in physical activity pre- and post-BC diagnosis decreased BC mortality, particularly for those who were inactive pre-diagnosis [11]. The type, frequency, and duration of activity best suited to lower BC risk is still unclear, however recent research in human epidermal growth factor receptor 2 (Her-2) BC cases found the greatest benefit in moderate to vigorous physical activity groups [12]. Some research suggests engaging in even recreational levels of exercise may still provide protective benefits against BC [13]. Exercise reduces BC risk by increasing anti-inflammatory cytokines and modulating hormones and genes involved in apoptosis and tumour cell signalling [11,14,15]. While other mechanisms may be involved, further research is needed.

Individually, existing evidence shows the consumption of specific dietary fats or the implementation of exercise programmes may influence BC development. However, the effects of exercise in combination with the consumption of various dietary fats have not been investigated. Her-2 BC is an aggressive form of human BC and is prevalent in 10–25% of women [16]. The MMTV-neu(ndl)-YD5 mouse model closely mimics human Her-2 positive BC and shares similar features such as Her2/neu overexpression, mammary gland tumour development and metastases, as well as treatment responses [16]. We have shown previously that dietary *n*-3 PUFA inhibits mammary tumour development in this mouse model [17,18]. Hence, the objective of this descriptive study was to investigate, for the first time, the combinatory effects of diet and exercise on mammary tumour outcomes in a model of Her-2 BC.

## 2. Materials and Methods

### 2.1. Animals and Diets

Each harem consisted of one male heterozygous MMTV-neu(ndl)-YD5 mouse (originally a gift from Dr. W.J. Muller, McGill University, Montreal, QC, Canada) and 3 female FVB wild type mice (Charles River, Laval, QC, Canada). Harems were randomly assigned to one of two diets (Research Diets, Inc., New Brunswick, NJ, USA): 10% *w*/*w* safflower oil, *n*-6 PUFA-enriched (n = 17, cat # D04092701) or 3% *w*/*w* menhaden fish oil + 7% *w*/*w* safflower oil, *n*-3 PUFA-enriched (n = 20, cat # D04092703). These diets are isocaloric and physiologically relevant based on our previous work [16,17,18]. Diet macronutrient composition was provided by the manufacturer (Table A1) and fatty acid composition was confirmed by gas chromatography (Table 1). Mice were provided ad libitum access to diet and double-distilled water. Offspring were weaned at 3 wks of age and genotyped for the MMTV-neu(ndl)-YD5 gene by polymerase chain reaction using the following primers: 5′-TTCCGGAACCCACATCAGGCC-3′ (forward) 5′-GTTTCCTGCAGCAGCCTACGC-3′ (reverse) as previously described [18]. Female offspring positive for the MMTV-neu(ndl)-YD5 gene were selected from a total of 15 litters (2–3 transgenic females per litter), maintained on maternal diets and allocated by simple randomization to either a sedentary (control) group or to the group assigned the exercise protocol, thus creating four experimental groups: (1) safflower sedentary (SS, n = 8), (2) safflower exercise (SE, n = 9), (3) menhaden fish oil sedentary (FS, n = 8) and (4) menhaden fish oil exercise (FE, n = 12). Male offspring were excluded from the study. Mice were housed together in ventilated cages with a maximum number of 4 mice per cage. Sedentary and exercised mice were housed separately. Investigators conducting the study and performing analyses were not blinded to treatment allocations. Starting at 3 wks of age, female mice were checked daily for vaginal opening, a marker of puberty onset. Body weights and food intakes were recorded weekly. At 20 wks of age, mice were euthanized by carbon dioxide asphyxiation, followed by cervical dislocation. All experimental procedures were approved by the University of Guelph’s Animal Care Committee (Animal Utilization Protocol # 4417, 20 January 2020).

### 2.2. Exercise Protocol

Further, 3 wk old offspring were randomly assigned to either an exercise protocol or remained sedentary (control) for each diet (n = 8–12 per group). At 7 wks of age, animals in the exercise group were acclimated to the treadmill for 15 min a day for 3 days a wk (Monday (M), Wednesday (W), and Friday (F)). During acclimation, the treadmill was not turned on. The exercise intervention began at 8 wks of age for a period of 4 wks and consisted of five, 45 min exercise sessions a wk (once per day). The treadmill was set to a 5° incline and speed was set to 10 m/min at wk 8 and increased by 2 m/min, each wk, up to 16 m/min at wk 11. At this speed, mice were able to stay on the treadmill continuously. If mice stopped running, they were gently tapped on the rear with a rubber policeman to enforce movement. Mice exercising within this range of speed are at 74–80% maximal oxygen consumption (VO_2_ max) [19] which is considered to be vigorous intensity exercise [20].

### 2.3. Mammary Tumour Measurements and Tissue Collection

**Figure 1:**
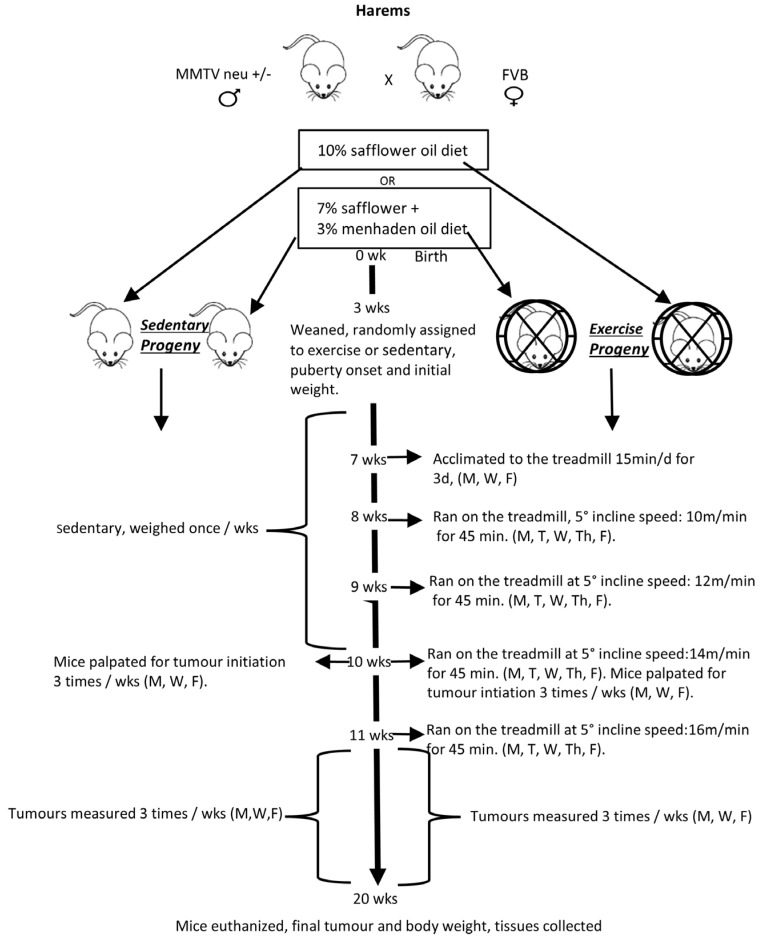
Experimental Design. Timeline of experiment from 0 to 20 wks with sedentary mice on the left side of the timeline and exercised mice on the right. Progeny was maintained on the parental diet throughout the study. M, T, W, Th, F are the days of the wk, Monday, Tuesday, Wednesday, Thursday and Friday, respectively.

Starting at 10 wks of age, equivalent to a woman in her twenties, tumours were palpated and measured using a digital calliper as described previously [16]. When a new tumour was detected, measurements were taken 3 times per wk until the mouse was terminated at 20 wks of age. Tumour volume was calculated using V = [length × (width^2^)]/2. If tumour dimensions exceeded 17 mm in length or width, or were more than 5000 mm^3^, mice were terminated prior to the 20 wk endpoint. This criterion was established *a priori*. No mice in this study were terminated early. Mice were terminated if in proestrus, estrus, or metestrus stages of the estrous cycle. If in the diestrus stage, termination was delayed for a maximum of 2 days to control for hormonal fluctuations. Estrous cycle stage was determined *via* vaginal flush as described previously [17]. At termination, the mouse pelt was removed with tumours attached and final tumour measurements were recorded. Subsequently, tumours were removed, weighed, and stored at −80 °C for future fatty acid analyses. An outline of the experimental design is provided in Figure 1.

### 2.4. Fatty Acid Analyses by Gas Chromatography

Lipid composition of diet and tumours was determined by gas chromatography (GC) as described previously [16,17,18]. In brief, lipids were extracted from 0.1 g of diet or 0.05 g of mammary gland tumours via the Folch Method [21]. Mammary gland tumours were randomly selected from 6 mice per group (also a random selection) and analyzed for phospholipid species by thin layer chromatography (TLC) as previously described [16,17,18]. Briefly, samples were spotted on H-plates (EMD Chemicals, Mississauga, ON, Canada, Cat #5721-7) to separate phospholipid species. Bands corresponding to phosphatidylcholine (PC) and phosphatidylethanolamine (PE) were collected and methylated with 14% boron trifluoride–methanol (BF3-MeOH, Fisher, Ottawa, ON, Canada, Cat #402760010). For diet analysis, samples were saponified with potassium hydroxide and methylated with 14% BF3-MeOH as previously described [22]. Fatty acid methyl esters were separated on a DB-FFAP fused-silica capillary column (15 m, 0.1 m film thickness, 0.1 mm i.d.; Agilent, Mississauga, ON, Canada, Cat # 127-32H2) and quantified on an Agilent 6890 gas chromatograph. Peaks were identified by retention times of fatty acid methyl ester standards (Nu-Chek-Prep, Elysian, MN, USA, Cat# GLC-463,) using EZchrom Elite version 3.2.1 software. Fatty acid results are expressed as percent composition of all fatty acid species.

### 2.5. Statistical Analysis

SAS version 9.4 (SAS Institute, Cary, NC, USA) was used for all statistical analyses. The upper limit of probability for statistical significance was set at *p* ≤ 0.05. A one-way analysis of variance (ANOVA) was applied to fatty acid data followed by Duncan’s Multiple Range test. Data sets that failed any of the four SAS tests for normality were subjected to Kruskal–Wallis testing for multiple-group comparisons followed by Wilcoxon two-sample rank sums, if justified by statistical significance. A two-way ANOVA was conducted to detect differences in tumour weight. A repeated measures analysis was applied to mouse bodyweight, diet consumption, tumour volume, and tumour number over the 20 wk experimental period. Log-rank test was used to determine the difference in proportion of tumour-free mice. Additionally, a Student’s *t*-test was performed to compare puberty onset between the diets. For tumour-free status, potential litter effects were analyzed using the Likelihood Ratio Test for Frailty, resulting in a litter variance of 0.0001, demonstrating that the 2–3 pups per litter used in the present study does not contribute to outcomes; thus, individual pups were used as statistical units [23]. All data is shown as mean + standard deviation (S.D.).

## 3. Results

### 3.1. Diet Intake and Body Weight

The level of dietary fat was chosen to compare against previously tested diets containing 10% safflower oil [16,17,18,22]. The 3% menhaden oil diet contains a physiologically relevant amount of eicosapentaenoic acid (EPA) and docosahexaenoic acid (DHA). Based on previous work [16], the average intake of food per mouse was ~2 g/day and therefore provided mice with 7.8 mg EPA and 6.8 mg DHA per day, or a combined 1.6% total daily energy. A traditional Japanese diet contains 1–2% of daily energy as EPA and DHA, translating to about 3.5 g of EPA and DHA/day based on a recommended 2000 kcal/day diet [24].

**Figure 2:**
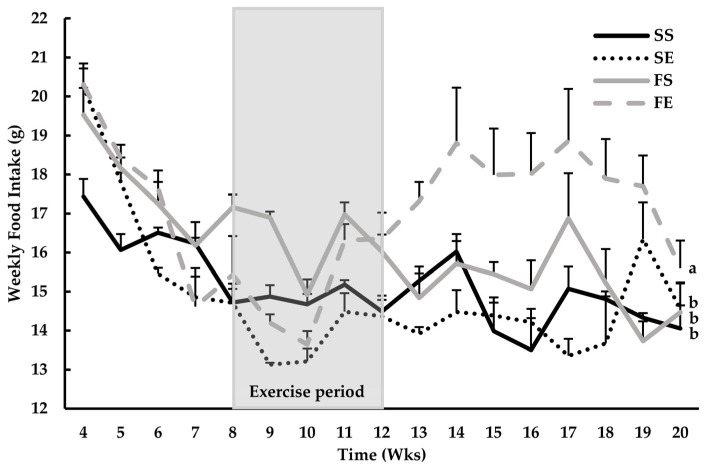
Diet consumption of mice over 20 wk experimental period. Mean + S.D. Lines not sharing a letter are significantly different according to repeated measures ANOVA (*p* < 0.0001).

**Figure 3:**
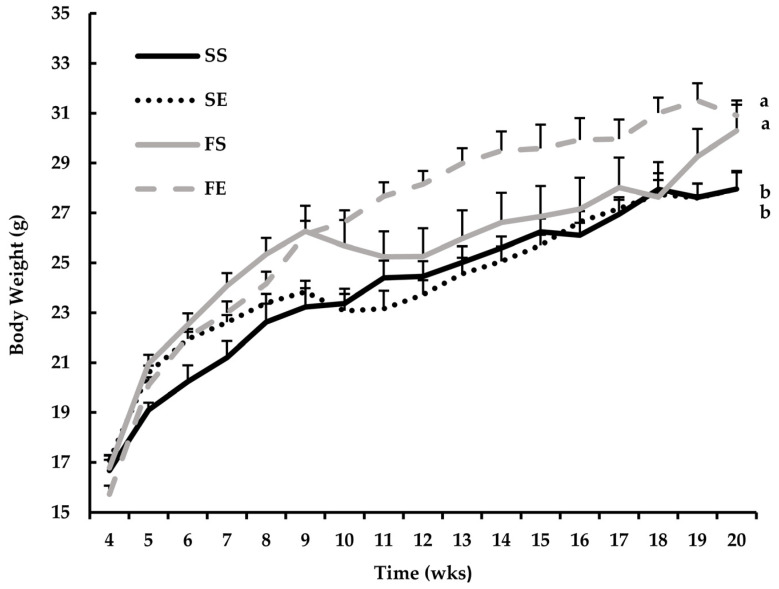
Mouse body weights over 20 wk time period. Mean + S.D. Lines not sharing a letter are statistically different according to a repeated measures ANOVA (*p* < 0.001). Groups are identified as safflower sedentary (SS, n = 8), safflower exercise (SE, n = 9), menhaden sedentary (FS, n = 8), and menhaden exercise (FE, n = 12). Shaded area highlights the 4-wk exercise period (wks 8–12).

Mice in all groups ate 2–3 g of food per day. Overall food intake (Figure 2) between safflower groups was not different; however, the FE group consumed more food than FS, SS or SE mice (*p* < 0.0001). This is more apparent after the exercise period (shaded in grey) where the food consumption of the FE group rises rapidly. For mouse body weights, both menhaden groups were heavier than the safflower groups throughout the 20 wk experimental period (*p* < 0.001, Figure 3). It is important to note, all groups had similar initial body weights with average initial weights of 16.7, 17.1, 16.1, and 16.8 g for SS, SE, FS, and FE respectively. Exercise (grey shaded area) had no effect on the safflower group but the FE group increased body weight from the first wk of the exercise period and consistently increased in body weight until the end of the study. This increase in body weight is reflective of the increase in food consumption of the FE mice. Final body weights were lower in mice fed the safflower diet compared to menhaden-fed mice (*p* = 0.0215).

Groups are identified as safflower sedentary (SS, n = 8), safflower exercise (SE, n = 9), menhaden sedentary (FS, n = 8), and menhaden exercise (FE, n = 12). The shaded area highlights the 4 wk exercise period (wks 8–12).

### 3.2. Puberty Onset

**Figure 4:**
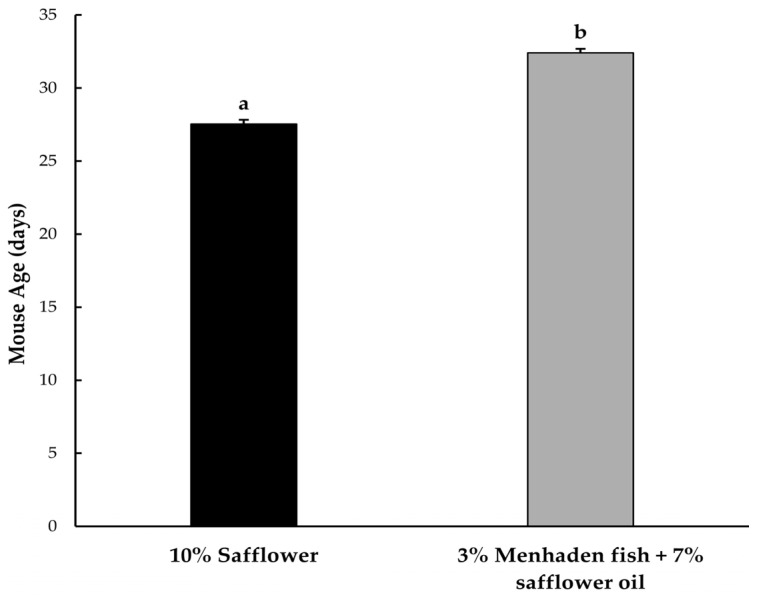
Puberty onset in MMTV female mice. Means + S.D. n = 17 for 10% safflower diet and n = 20 for 3% menhaden fish + 7% safflower oil diet. Bars with different letters are significantly different by Student’s *t*-test (*p* = < 0.0001).

Puberty onset is measured by the timing of the vaginal opening. Delay in puberty onset has been associated with reduced risk of developing BC [6,9]. In this experiment, puberty onset was delayed in the menhaden-fed mice compared to the safflower group (*p* < 0.0001) (Figure 4). This finding is consistent with previous studies on BC and *n*-3 PUFA [16,17,18].

### 3.3. Primary Tumour Outcomes

#### 3.3.1. Tumour Volume

**Figure 5:**
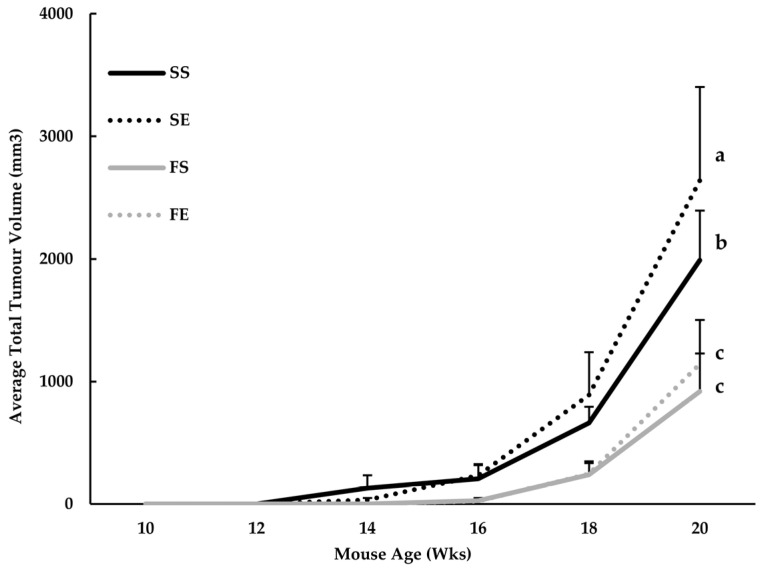
Tumour volume over time. Mean + S.D. Lines with different letters are significantly different by repeated measures ANOVA (*p* < 0.001). Groups are identified as safflower sedentary (SS, n = 8), safflower exercise (SE, n = 9), menhaden sedentary (FS, n = 8), and menhaden exercise (FE, n = 12).

Tumour volume was measured and calculated 3 times/wk starting at 10 wks of age (Figure 5). Mice fed the *n*-6 PUFA-enriched diet had consistently larger tumours throughout the experiment as seen in other studies [16,17,18,22]. At 20 wks of age (endpoint), mice fed the safflower diet (SS and SE) had tumours ~2.5 times larger than the menhaden fed mice (FS and FE). There was no difference in tumour volume between FS and FE groups. However, the SE group had significantly larger tumours than their sedentary counterparts (*p* < 0.001).

#### 3.3.2. Tumour Multiplicity

**Figure 6:**
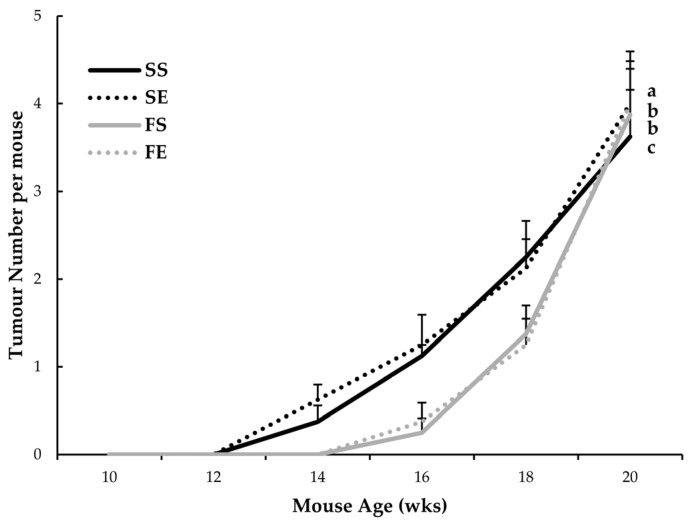
Number of tumours per mouse (multiplicity) over 20 wk time period. Mean + S.D. Lines not sharing a letter are statistically different by repeated measures ANOVA (*p* < 0.0001). FS and FE groups were not different from each other. Groups are identified as safflower sedentary (SS, n = 8), safflower exercise (SE, n = 9), menhaden sedentary (FS, n = 8), and menhaden exercise (FE, n = 12).

Tumour numbers were counted three times/wk starting at 10 wks of age (Figure 6). Throughout the experiment, mice fed the *n*-6 PUFA-enriched diet had consistently more tumours than *n*-3 PUFA-fed mice (*p* < 0.0001) as seen in other studies [16,17,18,22]. However, the SE group developed the most tumours by the 20 wk endpoint.

#### 3.3.3. Final Tumour Weight

**Figure 7:**
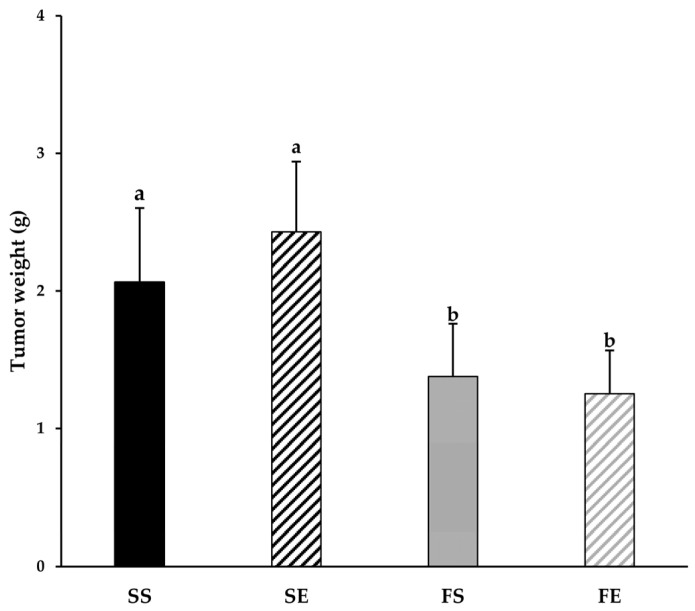
Total weight of all tumours combined per mouse. Mean + S.D. Bars not sharing a letter are different *p* ≤ 0.05 by 2-way ANOVA. The main effects of exercise and diet are shown. There was a main effect of diet (*p* = 0.0256) but no effect of exercise (*p* = 0.8624) and no interaction (*p* = 0.5193). Groups are identified as safflower sedentary (SS, n = 8), safflower exercise (SE, n = 9), menhaden sedentary (FS, n = 8), and menhaden exercise (FE, n = 12).

All tumours were collected and weighed at euthanasia (Figure 7). Tumours of the *n*-3 PUFA groups (FS and FE) weighed significantly less than those from the *n*-6 PUFA groups (SS and SE, *p* = 0.0256) and this is in line with similar findings from our research [16,17,18,22]. Exercise had no effect on final tumour weight in either diet group (*p* = 0.8624).

#### 3.3.4. Tumour-Free Status

**Figure 8:**
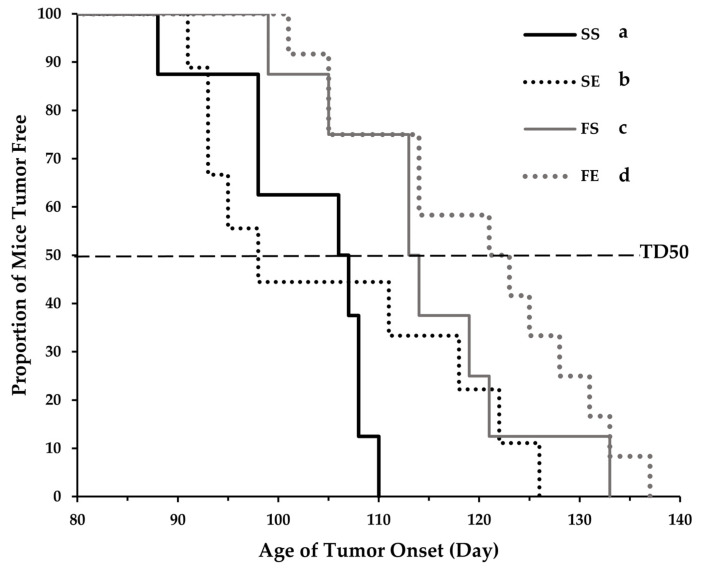
Tumour-free status of mice over 20 wks. Lines not sharing a letter are significantly different according to Log-rank test (*p* < 0.001). TD50, the median age when mammary tumours are present in 50% of the mice. Groups are identified as safflower sedentary (SS, n = 8), safflower exercise (SE, n = 9), menhaden sedentary (FS, n = 8), and menhaden exercise (FE, n = 12).

The TD_50_ threshold is the median age when mammary tumours are detected in 50% of the mice. Mice fed *n*-3 PUFA demonstrated longer tumour-free status than *n*-6 PUFA groups as seen in previous studies [16,22]. Importantly, the FE group demonstrated delayed tumour development by the greatest amount (123 days) compared to 113 days for FS and 117 days for SS (*p* < 0.001). Exercising mice fed *n*-3 PUFA (FE) experienced a ~24% longer period of tumour-free status compared to sedentary mice fed *n*-6 PUFA (SS) and ~10% longer tumour-free status than their sedentary counterparts (FS). In contrast, SE animals demonstrated the shortest tumour-free status with 50% of the mice developing tumours by 98 days (Figure 8).

#### 3.3.5. Fatty Acid Analysis

**Figure 9:**
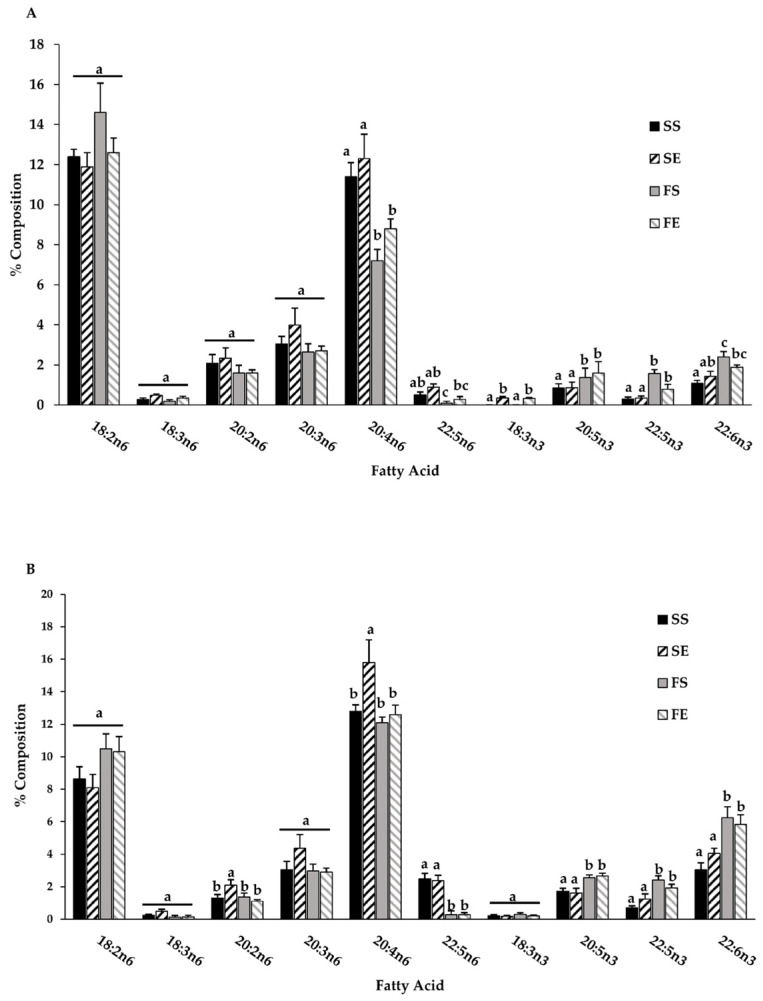
Fatty acid composition of mouse tumours. Mean + S.D. Percent composition of major *n*-6 and *n*-3 PUFA in PC (**A**) and PE (**B**) phospholipid fractions in 20 wk old mouse mammary gland tumour tissue. Different letters denote significant (*p* ≤ 0.05) differences between means by one-way ANOVA followed by Duncan’s Multiple Range Test, n = 6 per diet group. Complete fatty acid profiles of each phospholipid fraction can be found in Table A2 and Table A3. Groups are identified as safflower sedentary (SS, n = 8), safflower exercise (SE, n = 9), menhaden sedentary (FS, n = 8), and menhaden exercise (FE, n = 12).

The major *n*-6 and *n*-3 PUFA for phosphatidylcholine (PC) and phosphatidylethanolamine (PE) in mammary tumours is shown in Figure 9. A more detailed list of all fatty acids for these phospholipid species can be found in Table A2 and Table A3. In both PC and PE fractions, mice fed *n*-3 PUFA (FS and FE) had higher levels of EPA, docosapentaenoic acid (DPA, 22:5n3) and DHA and lower levels of arachidonic acid (AA) and DPA (22:5n6) compared to *n*-6 PUFA-fed mice (SS and SE). These results are comparable to other BC studies using the same diets in MMTV-neu (ndl) YD5 mice [16,17,18,22]. Exercise had no effect on lipid profiles except for α-linolenic acid (ALA) in the PC species where both SE and FE groups had higher ALA levels.

## 4. Discussion

The present study compares the effects of dietary *n*-3 and *n*-6 PUFA in combination with exercise on mammary tumour development. Although there is both epidemiological and experimental evidence supporting the beneficial effects of various dietary fats or exercise in BC [3,4,5,7,11,12,13,16,17,18,22] there is surprisingly a gap in research investigating the combined effects of specific dietary fats and exercise on BC development. Using the MMTV-neu (ndl)YD5 transgenic mouse model of Her-2 BC, the present study demonstrated that exercise combined with *n*-3 PUFA kept mice tumour-free for a longer period of time whereas exercise combined with *n*-6 PUFA exhibited the opposite effect (Figure 8). Further, exercise combined with an *n*-6 PUFA-enriched diet produced the largest and greatest number of tumours compared to their sedentary counterparts or to either of the *n*-3 PUFA groups. Similar to previous work [16,17,18,22], *n*-3 PUFA-fed mice had better tumour outcomes than mice fed *n*-6 PUFA. Therefore, lifelong exposure to dietary fat remains a primary determinant of mammary tumour outcomes, but exercise influences the time course of tumour development and may have additional positive effects if combined with an *n*-3 PUFA-enriched diet.

### 4.1. Role of Dietary Fat in Modulating Mammary Tumour Outcomes

This study demonstrates that lifelong exposure to dietary fat modulates tumourigenesis in BC. Most notably, menhaden oil (FS and FE) reduced tumor volume (Figure 5) and final tumour weight (Figure 7) and delayed puberty onset (Figure 4) compared to safflower oil (SS and SE). These findings confirm results from previous research which show diets high in *n*-3 PUFA are protective against mammary tumorigenesis whereas diets high in *n*-6 PUFA are tumour promoting [16,17,18]. A recent meta-analysis investigating *n*-3 PUFA consumption among Asian patients with BC confirmed the protective effects of *n*-3 PUFA on BC [24]. The beneficial effects of *n*-3 versus *n*-6 PUFA in the current study also coincide with the finding that a high *n*-3/*n*-6 ratio is best for BC management and treatment [25].

### 4.2. Role of Dietary Fat and Exercise on Tumour Outcomes

The present study suggests that exercise impacts the time course of tumour formation and the outcome is dependent on diet. Time course analysis (survival curve) shows exercise combined with *n*-3 PUFA (FE) kept mice tumour-free for a 10% longer period of time than their sedentary counterparts (FS) and 24% longer than SS (Figure 8). Exercise did not affect final tumour outcomes (volume (Figure 5), number (Figure 6), or weight (Figure 7)) in the FS and FE groups Exercise combined with *n*-6 PUFA (SE) had the most tumour-bearing mice in the shortest period of time, with mice bearing tumours 10 days sooner than their sedentary counterparts (SS) and 15 and 25 days sooner than FS and FE, respectively. Furthermore, SE had the greatest tumour volume (Figure 5) and number (Figure 6) than any of the other three groups. The acceleration of tumour development by *n*-6 and exercise is unexpected and suggests additional research is needed to determine in what conditions exercise may be warranted. We speculate that while exercise is generally considered positive, exercise exerts physical stress and inflammation on the body; thus, in specific conditions it may promote rather than inhibit disease progression.

The TD50 line in Figure 8, the time when 50% of the mice are tumour-bearing, provides interesting results if related to the compression of morbidity hypothesis [26]. The compression of morbidity hypothesis attests to try to find life factors that keep the best quality of life for the longest period of time. For example, being ill for the shortest period of time at the end of life. In this study, all mice developed tumours by the 20 wk endpoint. However, the FE mice were tumour-free longer than the other three groups, and thus had a longer disease-free life. Considering the time-course, exercise appears to add additional protective effects against tumour formation when combined with an *n*-3 PUFA diet. Exercise and *n*-3 PUFA are protective against BC by activating similar biological pathways. Most notably, exercise and *n*-3 PUFA can modify energy metabolism, epigenetics, inhibit tumour microenvironment inflammation and regulate immune responses via cytokine expression and immune cells [14,15,26,27]. The finding of a potential combinatory effect of exercise and *n*-3 PUFA over time is preliminary and warrants further investigation as the same combinatory effect was not observed in specific outcome measures in this study. What our work may have revealed is the need to differentiate outcomes and measures that reflect lifespan versus healthspan. Considering only final outcomes at one timepoint provides an incomplete picture when there is a growing interest in both lifespan and quality of life. Quality of life considerations are reflected in the growing interest in healthspan and compression of morbidity research which focuses on how interventions lengthen the number of years of life that are disease-free, which may not necessarily be accompanied by an increase in lifespan [23,28].

Studies investigating diet and exercise together are limited. Especially for marine-derived *n*-3 PUFA or *n*-6 PUFA-enriched diets. However, there are a few studies investigating the effects of soy (plant-based source of ALA) and exercise which found comparable results and may provide hypotheses for our findings. For example, Wang et al. investigated daidzein, a naturally occurring isoflavone found in soybeans and legumes, with exercise in BALB/c mice transplanted with BC cells. Results showed exercise alone or daidzein alone inhibited tumour growth in mice to a different degree. However, when exercise and daidzein were combined there was a synergistic inhibition on tumour growth, compared with the control. The combination of exercise and daidzein synergistically mobilized natural killer cells through epinephrine and interleukin-6 (IL-6) upregulation. Furthermore, exercise combined with daidzein induced apoptosis in cancer cells via the Fas/FasL-initiated signalling pathway [29]. A similar study, by Kwon et al., investigated genistein (GEN), another isoflavone found in soy, with exercise on mice with MG tumours and found exercise to be synergistic with GEN in delaying tumour initiation and growth. The combination of exercise and GEN increased the expression of apoptosis markers and increased the M1/M2 macrophage ratio. This was accomplished by decreasing M2 polarization through the Jak1/STAT6 signalling pathway [30].

In contrast to the protective effects of *n*-3 PUFA, *n*-6 PUFA promote tumour growth, are pro-inflammatory and alter gene expression to activate cell proliferation [16]. In the current study, mice fed *n*-6 PUFA had the largest and greatest number of tumours and formed tumours faster than *n*-3 PUFA-fed mice. Exercise combined with an *n*-6 PUFA diet further exacerbated tumour development compared with the *n*-6 diet in the sedentary state. To our knowledge, there are no previous studies that have investigated the effects of *n*-6 PUFA diet and exercise on BC outcomes. However, a prostate study by Veras et al. [31] showed that obese rats fed a high-fat diet combined with aerobic exercise had increased prostatic inflammation by increasing IL-6 and tumour-inflammatory cytokines necrosis factor alpha (TNF α) via similar mechanisms as *n*-6 PUFA to activate tumour promoting inflammatory cytokines [32]. The unexpected combinatory effect of *n*-6 PUFA and exercise resulting in pro-tumour effects warrants further investigation given that the North American diet is high in *n*-6 PUFA and lifestyle modification including exercise is recommended for prevention and disease management of cancer.

In this study, the timing of exercise (just before tumour development) was specifically chosen to investigate the effects of acute exercise on tumour prevention. It is also important to note that the exercise timing, in this study, reflects women who often stop exercising once BC is diagnosed or after tumour treatment begins due to multiple barriers [7]. However, both timing and exercise intensity may play a role in BC development. There are many studies investigating multiple types and intensities of exercise and cancer. A study of voluntary wheel running in a mouse model found that, while early tumour development was attenuated through exercise, this anti-tumour effect was absent in later stages [33]. In another study of exercise and BC, BALB/c mice undertook eight wks of aerobic treadmill exercise, beginning before tumour formation. In this case, exercise reduced tumour formation throughout the study by reducing miR-21 gene expression and increasing hypoxia-inducible factor-1α (HIF-1α), caspase-9 and caspase-3 expression, thereby suppressing apoptosis and reducing angiogenesis [34]. Finally, in a tumour mouse model, it has been shown that stretching for 10 min a day decreases tumour size by 52% through an increase in lipid mediators resolvin D1 (RvD1) and resolvin D2 (RvD2). RVD1 and RVD2 have significant anti-inflammatory properties and are derived from the *n*-3 PUFA DHA in tissues [19]. In summary, many studies have investigated the effects of exercise on mammary tumour development. These studies range in exercise intensity, amount and timing. Despite these differences, all studies show exercise to delay tumour development or reduce tumours through immune pathways or gene modulation. A less aggressive form of exercise such as stretching or walking may be more attainable in women who are diagnosed with BC; however, more studies on exercise intensity and timing are needed.

### 4.3. Study Limitations

The results of this pilot study are relevant to women with Her-2 BC as they provide insight into how diet and exercise may influence mammary tumour development. The scientific literature has numerous studies investigating either diet or exercise independently, but few study these factors in tandem. Thus, this initial descriptive study is important in investigating the potential combinatory effects of diet and exercise and to determine whether additional mechanistic studies are warranted.

The MMTV-neu-(ndl) YD5 mouse model used mimics human Her-2 positive BC, as over-expression of Her-2 is associated with an invasive and highly metastatic phenotype in women with BC [35]. This mouse model develops tumours rapidly with maximum development in 20 weeks. Although this model is used to highlight clinical parallels experienced by women with this BC subtype, this mouse model used may have been too aggressive or the duration of the exercise intervention may have been too short to observe all potential effects of exercise throughout the experimental period. As a continuation of this study, a less aggressive mouse model, such as MMTV-neu, where tumours develop over the course of a year, should be considered to observe the effects of lifelong exercise on mammary tumour outcomes.

In this study, diet intake and body weights differ between groups which may affect energy balance and adiposity, factors known to influence breast cancer risk and survivorship in women. The absence of body composition and energy intake measurements should be considered when interpreting the results of this study. Future studies using a pair-fed design will help clarify the contribution of energy balance to tumour development.

Finally, the sample size of 8–12 mice per group is modest and the exploratory findings herein should be interpreted with this factor in mind.

## 5. Conclusions

In conclusion, the present study shows that diet plays a major role in mammary tumour development and potentially may be further influenced by exercise. To our knowledge, this is the only study to investigate the combined effects of exercise with marine-based *n*-3 PUFA on mammary tumour development in mice and warrants further investigation. Lastly, our work highlights the need to consider temporal changes in addition to terminal outcomes in the growing field of healthspan research.

## Figures and Tables

**Table 1:** Fatty acid composition of modified AIN-93G rodent diets ^1^.

Fatty Acid (% of Total Fatty Acids)	10% Safflower Oil	3% Menhaden Fish + 7% Safflower Oil
12:0	0.04	0.08
14:0	0.24	2.69
16:0	6.92	10.18
16:1c9	0.15	4.02
18:0	2.59	2.87
18:1c9	15.27	13.86
18:1c11	0.80	1.48
18:2n6	72.00	52.04
18:3n6	0.11	0.16
18:3n3	0.31	0.87
18:4n3	0.13	0.90
20:0	0.36	0.36
20:1c11	0.22	0.49
20:2n6	0.05	0.12
20:3n6	0.00	0.10
20:4n6	0.00	0.50
20:3n3	0.00	0.20
20:5n3	0.05	4.22
22:0	0.30	0.27
22:1n9	0.02	0.09
22:2n6	0.00	0.28
22:4n6	0.11	0.14
22:5n3	0.00	0.75
24:0	0.14	0.02
22:6n3	0.00	3.10
24:1	0.17	0.23
Total %	100	100
% Saturated	10.59	16.46
% Monounsaturated	16.63	20.16
% *n*-3 Polyunsaturated	0.49	10.05
% *n*-6 Polyunsaturated	72.28	53.33

^1.^ Values are means and represent percent of total fatty acids in each diet. Analyses were performed in duplicate and fatty acid composition was determined by gas chromatography.

## Data Availability

The original contributions presented in this study are included in the article. Further inquiries can be directed to the corresponding authors.

## Abbreviations

The following abbreviations are used in this manuscript:

wks	weeks
PUFA	Polyunsaturated fatty acids
TD50	Tumour-free status
BC	Breast cancer
Her-2	Human epidermal growth factor receptor 2
SS	Safflower sedentary
SE	Safflower exercise
FS	Menhaden fish oil sedentary
FE	Menhaden fish oil exercise
VO_2max_	Maximal oxygen consumption
GC	Gas chromatography
TLC	Thin layer chromatography
PC	Phosphatidylcholine
PE	Phosphatidylethanolamine
BF3-MEOH	Boron trifluoride-methanol
ANOVA	Analysis of variance
EPA	Eicosapentaenoic acid
DHA	Decosahexaenoic acid
ALA	a-linolenic acid
IL-6	Interleukin-6
GEN	Genistein
HFD	High-fat diet
TNF-a	Tumour necrosis factor-a
HIF-1a	Hypoxia-inducible factor 1a
RvD1	Resolvin D1
RvD2	Resolvin D2

## Appendix A

**Table A1:** Composition of modified AIN-93 G rodent diets (Research Diets) ^1^.

	10% Safflower Oil (D04092701)		3% Menhaden + 7% Safflower Oil (D04092703)	
**Macronutrient**	g %	kcal %	g %	kcal %
Protein	21	20	21	20
Carbohydrate	60	58	60	58
Fat	10	22	10	22
Total		100		100
Kcal/g	4		4	
**Ingredient**	g	kcal	g	kcal
Casein	200	800	200	800
L-cystine	3	12	3	12
Corn starch	337	1347	337	1347
Maltodextrin 10	132	528	132	528
Sucrose	100	400	100	400
Cellulose, BW200	50	0	50	0
Safflower Oil	97	873	68	611
Menhaden Oil	0	0	29	262
Flax Oil	0	0	0	0
Olive Oil	0	0	0	0
Lard	0	0	0	0
t-Butylhydroquinone	0.02	0	0.02	0
Mineral mix S10022 G	35	0	35	0
Vitamin mix V10037	10	40	10	40
Choline bitartrate	2.5	0	2.5	0
**Total**	966	4000	966	4000

^1^ AIN-93G rodent diets (Research Diets catalogue number).

**Table A2:** Percent composition of fatty acids in phosphatidylcholine fraction of mouse mammary gland tumour ^1^.

Fatty Acids	SS	SE	FS	FE
14:0	1.46 ± 0.23	1.19 ± 0.31	1.43 ± 0.33	1.71 ± 0.50
15:0	0.38 ± 0.12	0.35 ± 0.10	0.42 ± 0.10	0.44 ± 0.08
16:0	28.3 ± 1.78	27.5 ± 2.44	28.1 ± 1.98	28.9 ± 2.16
18:0	9.06 ± 2.17	8.33 ± 1.31	9.45 ± 2.38	8.60 ± 0.87
19:0	0.33 ± 0.18	0.56 ± 0.18	0.40 ± 0.14	0.42 ± 0.24
20:0	0.42 ± 0.42	0.46 ± 0.46	0.46 ± 0.40	0.46 ± 0.41
22:0	0.63 ± 0.31	0.62 ± 0.17	0.64 ± 0.64	0.66 ± 0.45
24:0	0.50 ± 0.23	0.43 ± 0.24	0.55 ± 0.20	0.61 ± 0.27
Total SFA	41.0 ± 2.36	39.4 ± 2.23	41.4 ± 2.55	41.8 ± 1.76
16:1c9	3.23 ± 1.01	3.05 ± 0.50	3.18 ± 1.34	3.69 ± 0.68
18:1c9	13.0 ± 0.87	12.5 ± 1.63	12.6 ± 2.19	13.6 ± 1.16
18:1c11	5.14 ± 0.82	5.17 ± 1.01	5.63 ± 1.42	6.04 ± 0.60
20:1c11	0.98 ± 0.39	0.98 ± 0.48	0.87 ± 0.23	0.90 ± 0.16
22:1n9	2.20 ± 0.88 ^ab^	1.36 ± 0.41 ^b^	2.60 ± 1.13 ^a^	1.29 ± 0.55 ^b^
24:1n9	0.53 ± 0.23	0.58 ± 0.22	0.50 ± 0.16	0.44 ± 0.21
Total MUFA	25.1 ± 2.75	23.6 ± 2.15	25.3 ± 4.64	26.0 ± 1.82
18:2n6	12.4 ± 0.94	11.9 ± 1.71	14.6 ± 3.61	12.6 ± 1.78
18:3n6	0.28 ± 0.18	0.48 ± 0.18	0.18 ± 0.20	0.34 ± 0.25
18:3n3 ^ŧ^	0.00 ± 0.00 ^a^	0.35 ± 0.17 ^b^	0.00 ± 0.00 ^a^	0.33 ± 0.09 ^b^
18:4n3	0.28 ± 0.23	0.28 ± 0.27	0.32 ± 0.20	029 ± 0.28
20:2n6	2.09 ± 1.05	2.34 ± 1.24	1.60 ± 0.93	1.55 ± 0.36
20:3n6	3.05 ± 0.91	3.99 ± 2.07	2.64 ± 1.00	2.70 ± 0.58
20:4n6	11.4 ± 1.71 ^a^	12.3 ± 2.99 ^a^	7.21 ± 1.40 ^b^	8.80 ± 1.21 ^b^
20:5n3 ^ŧ^	0.85 ± 0.21 ^a^	0.86 ± 0.28 ^a^	1.38 ± 0.46 ^b^	1.59 ± 0.58 ^b^
22:2n6	0.67 ± 0.42	0.90 ± 0.36	0.98 ± 0.59	0.73 ± 0.48
22:4n6	0.85 ± 0.43 ^a^	0.94 ± 0.22 ^a^	0.38 ± 0.37 ^b^	0.43 ± 0.24 ^b^
22:5n6	0.51 ± 0.34 ^ab^	0.89 ± 0.40 ^a^	0.09 ± 0.22 ^c^	0.28 ± 0.33 ^bc^
22:5n3 ^ŧ^	0.30 ± 0.26 ^a^	0.34 ± 0.33 ^a^	1.57 ± 0.61 ^b^	0.79 ± 0.15 ^b^
22:6n3	1.08 ± 0.38 ^a^	1.44 ± 0.58 ^ab^	2.40 ± 0.67 ^c^	1.89 ± 0.26 ^bc^
Total PUFA	33.8 ± 2.39 ^b^	37.0 ± 1.74 ^a^	33.4 ± 2.66 ^b^	32.3 ± 1.83 ^b^
Total n6	31.3 ± 2.85 ^a^	33.7 ± 2.17 ^a^	27.7 ± 3.66 ^b^	27.4 ± 1.68 ^b^
Total n3 ^ŧ^	2.51 ± 0.69 ^b^	3.27 ± 0.99 ^b^	5.66 ± 2.72 ^a^	4.89 ± 0.55 ^a^
n6/n3 ^ŧ^	13.9 ± 6.27 ^a^	11.4 ± 4.45 ^a^	5.70 ± 2.1 ^b^	5.67 ± 0.69 ^b^

^1^ Within a row, values not sharing a letter are significantly different according to one-way ANOVA (*p* < 0.05) followed by Duncan’s Multiple Range Test. Values are expressed as mean ± S.D. (n = 6) for safflower sedentary (SS), safflower exercise (SE), menhaden fish oil sedentary (FS) and menhaden fish oil exercise (FE). ^ŧ^ Data did not conform to normal distribution and was analyzed by Kruskal–Wallis followed by Wilcoxon’s two-sample test.

**Table A3:** Percent composition of fatty acids in phosphatidylethanolamine fraction of mouse mammary gland tumour ^1^.

Fatty Acids	SS	SE	FS	FE
14:0	1.04 ± 0.48	0.92 ± 0.55	0.75 ± 0.45	1.24 ± 0.79
15:0	0.39 ± 0.19	0.31 ± 0.26	0.25 ± 0.18	0.31 ± 0.20
16:0	10.2 ± 1.18 ^ab^	8.39 ± 0.83 ^c^	9.07 ± 1.03 ^bc^	10.38 ± 0.72 ^a^
18:0	15.0 ± 1.10	13.1 ± 1.26	14.8 ± 2.76	14.0 ± 2.90
19:0	0.70 ± 0.11	0.62 ± 0.37	0.51 ± 0.31	0.70 ± 0.09
20:0	0.98 ± 0.83	1.34 ± 1.41	0.68 ± 0.75	0.89 ± 0.78
22:0	1.17 ± 067	1.38 ± 1.04	0.72 ± 0.48	1.17 ± 0.68
24:0	1.27 ± 0.99	0.80 ± 0.61	1.03 ± 0.98	1.02 ± 0.39
Total SFA	30.7 ± 1.54	26.9 ± 3.98	27.8 ± 3.58	29.7 ± 2.03
16:1c9	1.97 ± 0.71	2.50 ± 0.80	2.32 ± 0.80	3.00 ± 1.51
18:1c9	16.3 ± 0.60	16.6 ± 2.62	16.1 ± 3.70	17.7 ± 2.24
18:1c11	4.67 ± 1.60	4.62 ± 1.09	5.40 ± 2.10	5.93 ± 0.97
20:1c11	0.91 ± 0.24	1.40 ± 0.57	1.17 ± 0.52	0.86 ± 0.24
22:1n9	3.67 ± 1.00	2.85 ± 1.95	3.51 ± 2.45	2.72 ± 0.99
24:1n9	0.42 ± 0.20	0.40 ± 0.33	0.60 ± 0.24	0.55 ± 0.46
Total MUFA	28.0 ± 2.56	28.4 ± 1.68	29.1 ± 7.95	30.7 ± 4.31
18:2n6	8.63 ± 1.68	8.09 ± 1.85	10.5 ± 2.04	10.3 ± 2.29
18:3n6	0.23 ± 0.18	0.48 ± 0.30	0.13 ± 0.21	0.15 ± 0.21
18:3n3	0.22 ± 0.15	0.19 ± 0.11	0.30 ± 0.22	0.20 ± 0.16
18:4n3	0.78 ± 0.55	0.73 ± 0.50	0.70 ± 0.25	0.88 ± 0.43
20:2n6	1.30 ± 0.50 ^b^	2.10 ± 0.74 ^a^	1.36 ± 0.56 ^b^	1.07 ± 0.24 ^b^
20:3n6	3.05 ± 1.15	4.36 ± 1.90	2.96 ± 0.96	2.85 ± 0.58
20:4n6	12.8 ± 0.87 ^b^	15.8 ± 3.27 ^a^	12.1 ± 0.76 ^b^	12.6 ± 1.21 ^b^
20:5n3	1.73 ± 0.39 ^a^	1.62 ± 0.63 ^a^	2.55 ± 0.38 ^b^	2.67 ± 0.38 ^b^
22:2n6	0.83 ± 0.45	0.81 ± 0.43	0.42 ± 0.38	0.54 ± 0.42
22:4n6	2.29 ± 0.42 ^a^	2.12 ± 0.46 ^a^	0.95 ± 0.74 ^b^	0.52 ± 0.36 ^b^
22:5n6	2.49 ± 0.74 ^a^	2.37 ± 0.75 ^a^	0.29 ± 0.50 ^b^	0.28 ± 0.31 ^b^
22:5n3	0.70 ± 0.26 ^a^	1.24 ± 0.33 ^a^	2.41 ± 0.58 ^b^	1.93 ± 0.54 ^b^
22:6n3	3.04 ± 0.96 ^a^	4.04 ± 0.73 ^a^	6.24 ± 1.52 ^b^	5.83 ± 1.44 ^b^
Total PUFA	38.1 ± 3.13	44.0 ± 4.22	40.9 ± 3.43	39.8 ± 2.26
Total n6	31.6 ± 4.25 ^b^	36.1 ± 4.71 ^a^	28.7 ± 2.38 ^b^	28.3 ± 1.45 ^b^
Total n3	6.48 ± 1.27 ^a^	7.84 ± 1.19 ^a^	12.2 ± 1.87 ^b^	11.5 ± 2.31 ^b^
n6/n3 ^ŧ^	5.11 ± 1.54 ^a^	4.75 ± 1.23 ^a^	2.38 ± 0.32 ^b^	2.56 ± 0.61 ^b^

^1^ Within a row, values not sharing a letter are significantly different according to one-way ANOVA (*p* < 0.05) followed by Duncan’s Multiple Range Test. Values are expressed as mean ± S.D. (n = 6) for safflower sedentary (SS), safflower exercise (SE), menhaden fish oil sedentary (FS) and menhaden fish oil exercise (FE). ^ŧ^ Data did not conform to normal distribution and was analyzed by Kruskal–Wallis followed by Wilcoxon’s two-sample test.
